# Free water elimination tractometry reveals local and remote white matter alterations in diffuse gliomas

**DOI:** 10.1007/s11060-025-05370-w

**Published:** 2025-12-10

**Authors:** Daniel J. Zhou, Kelly Chang, Marc Jaskir, Kathryn A. Davis, Joel M. Stein, Nishant Sinha, Richard E. Phillips, Manuel Ferreira, Thomas J. Grabowski, Ariel Rokem

**Affiliations:** 1 Department of Neurology, University of Pennsylvania, 301 Hayden Hall 3320 Smith Walk, Philadelphia, PA 19104 USA; 2 Center for Neuroengineering and Therapeutics, University of Pennsylvania, Philadelphia, PA USA; 3 eScience Institute, University of Washington, Philadelphia, PA USA; 4 Department of Psychology, University of Washington, Philadelphia, PA USA; 5 Department of Neuroscience, University of Pennsylvania, Philadelphia, PA USA; 6 Department of Radiology, University of Pennsylvania, Philadelphia, PA USA; 7 Division of Hematology/Oncology, Department of Medicine, University of Pennsylvania, Philadelphia, PA USA; 8 Department of Neurological Surgery, University of Washington School of Medicine, Seattle, WA USA; 9 Department of Radiology, University of Washington, Seattle, WA USA; 10 Department of Neurology, University of Washington, Seattle, WA USA

**Keywords:** Glioma, White matter, Diffusion MRI, Tractometry, Free water elimination

## Abstract

**Purpose:**

To apply free water elimination (FWE) tractometry to a real-world clinical imaging dataset to quantify pathology-specific patterns of white matter involvement and peritumoral tissue alterations in diffuse gliomas.

**Methods:**

The University of California San Francisco Preoperative Diffuse Glioma MRI dataset was analyzed using FWE tractometry. Twenty major white matter tracts were reconstructed and each divided into 100 equidistant nodes. Direct tumor involvement was quantified across enhancing tumor, necrotic core, and edema regions. Remote white matter tissue properties were assessed through hemispheric asymmetry analysis of free water-corrected fractional anisotropy (FW-FA), mean diffusivity (FW-MD), and free water fraction (FWF) in non-tumor involved regions at standardized distances from radiological tumor margins.

**Results:**

459 patients with unilateral glioma were included (361 glioblastoma, 87 astrocytoma, 11 oligodendroglioma). Glioblastoma demonstrated greater direct white matter involvement in enhancing tumor and necrotic core compared to astrocytoma and oligodendroglioma (*q* < 0.001, *q* = 0.01, respectively). Beyond radiological tumor margins, glioblastoma and astrocytoma exhibited decreased FW-FA, while oligodendroglioma showed increased FW-FA (*q* = 0.008, *q* = 0.04, respectively). Distance-based analysis revealed that this effect was most prominent in the proximal peritumoral region and diminished with increasing distance from tumor margins.

**Conclusion:**

Using FWE tractometry on a large clinical repository, we identified distinct pathology-specific patterns of white matter alteration. Glioblastoma showed extensive direct involvement and peritumoral microstructural changes, while oligodendroglioma demonstrated relatively preserved white matter architecture near tumor margins. These patterns reflect expected biological differences and provide a reproducible framework for characterizing extent of white matter involvement, with potential applications in presurgical planning and understanding recurrence patterns.

**Supplementary Information:**

The online version contains supplementary material available at 10.1007/s11060-025-05370-w.

## Introduction

Adult-type diffuse gliomas are the most common primary malignant brain tumors and remain difficult to treat [1]. Understanding how different glioma subtypes interact with brain white matter could contribute to surgical planning and predicting recurrence patterns. However, systematic comparisons of white matter involvement patterns across glioma subtypes have been limited by the need for large cohorts with comprehensive molecular characterization. Histopathological studies have demonstrated that glioma cells disseminate faster along white matter bundles than through cortex, using myelinated fibers as scaffolds for invasion [2–5]. Tissue biopsy studies have revealed that viable tumor cells remain present beyond enhancing regions visible on neuroimaging and intraoperative surgical margins [6, 7]. Glioblastoma disrupts structural brain networks and favors recurrence along impacted white matter pathways, while lower grade gliomas demonstrate more variable infiltration patterns [4, 8–11].

Conventional diffusion tensor imaging (DTI) and tractography studies of glioma are limited by tumor-induced artifacts, including vasogenic edema, mass effect, and variable infiltration, that alter diffusion measures and impede tractography [12, 13]. Free water elimination (FWE) methods separate tissue-specific diffusion from free water contamination, enabling more accurate assessment of white matter tissue properties [14–16]. MRI-based tractometry samples diffusion metrics at standardized points along anatomically validated fiber bundles, offering a reproducible framework for quantifying white matter involvement across institutions [17, 18].

The University of California San Francisco Preoperative Diffuse Glioma (UCSF-PDGM) MRI dataset is one of the largest publicly available preoperative imaging repositories with standardized protocols, expert tumor segmentations, and comprehensive molecular characterization across gliomas [19, 20]. Using this dataset, we aim to: (1) quantify direct tumor involvement of white matter tracts across glioma subtypes, and (2) assess white matter tissue property changes along white matter tracts beyond radiologic tumor margins.

## Materials and methods

### Dataset and participants

The UCSF-PDGM dataset is a publicly available collection of preoperative imaging and clinical data from adult patients with diffuse gliomas [19]. The images include MRI T1 and T2 imaging, DTI, and segmented tumor components (enhancing tumor, necrotic core, and edema/infiltration regions). These protocols have been previously described; in brief, diffusion-weighted MRI was collected at 2 mm^3^ isotropic resolution, 55 directions, with b = 2,000 s/mm^2^ [21]. For white matter asymmetry analyses, we included patients with unilateral white matter tract involvement. Bilateral or midline tumors were excluded to avoid confounding asymmetry measurements. The dataset provided diagnoses labeled according to the WHO 2021 classification. After correspondence with the dataset authors, cases labeled “astrocytoma *IDH*-wildtype” were confirmed to represent tumors with incomplete molecular testing, therefore reclassified as Not Elsewhere Classified (NEC). To ensure diagnostic accuracy for pathology-specific analyses, we excluded NEC cases and focused on patients with definitive histopathologic and molecular diagnoses of glioblastoma, astrocytoma, or oligodendroglioma.

### Tractography and free water elimination

Diffusion images were preprocessed using QSIPrep, which performs motion correction, distortion correction, and denoising [22]. Tractography reconstruction, tractometry, and FWE analysis were performed as previously described, using the open-source pyAFQ software version 2.1, which relies on techniques implemented in the DIPY software [15, 17]. Tractography was performed in native subject space, and tract identification used pyAFQ’s anatomically-defined waypoint region of interest approach. Twenty major white matter tracts were reconstructed bilaterally, including the anterior thalamic radiation, arcuate fasciculus, posterior arcuate fasciculus, cingulate section of the cingulum bundle, corticospinal tract, inferior fronto-occipital fasciculus, inferior longitudinal fasciculus, superior longitudinal fasciculus, uncinate fasciculus, and vertical occipital fasciculus. Each tract was divided into 100 equidistant nodes along its length, standardizing sampling to equivalent anatomical positions across subjects with varying brain sizes and tract lengths.

**Fig. 1:**
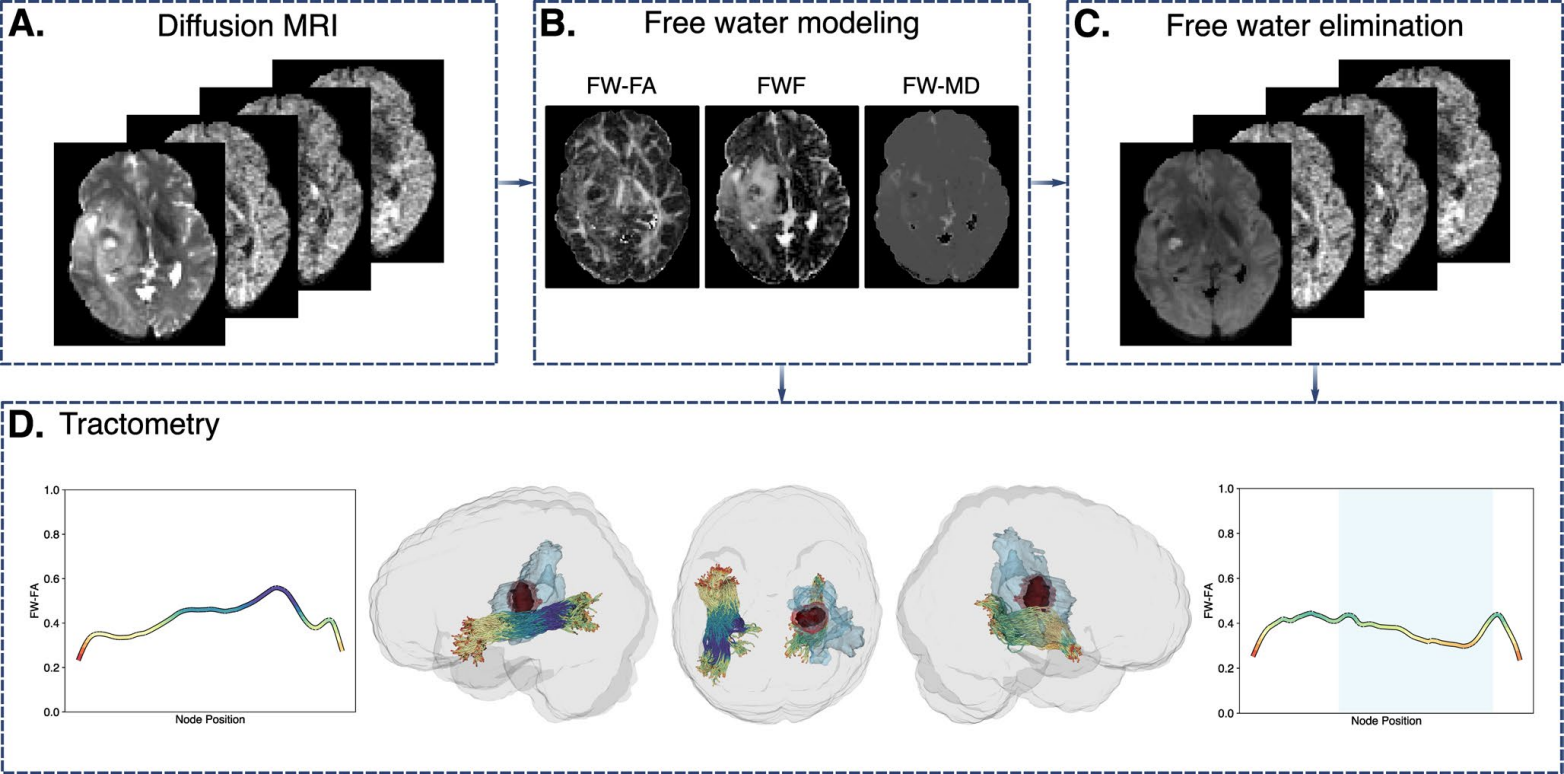
Free water elimination and tractometry pipeline. (**A**) Preprocessed diffusion MRI acquisitions. (**B**) Free water modeling produces free water-corrected (FW) fractional anisotropy (FW-FA), free water fraction (FWF), and mean diffusivity (FW-MD) metrics. (**C**) The free water model metrics are used to compute free water eliminated diffusion MRI values from the preprocessed diffusion images. (**D**) The free water eliminated images are used to create tracts and tract profiles. Left and right inferior longitudinal fasciculi are shown with corresponding tract profiles colored by FW-FA values. The black, red, and blue regions correspond to the enhancing tumor, necrotic core, and edema/infiltration regions, respectively. The blue background in the right longitudinal fasciculus represents the nodes considered involved (> 5%) in the edema region

Tract profiles were generated by sampling diffusion metrics along the trajectory of each tract. Because the data had measurements with only one diffusion weighting, we used an implementation of a free-water DTI model that uses spatial continuity constraints to regularize model fit [16, 23]. FWE was applied to separate tissue-specific diffusion from free water contamination, yielding FW-corrected fractional anisotropy (FA), mean diffusivity (MD), and free water fraction (FWF) metrics (Fig. 1).

All reconstructed tracts underwent automated quality control procedures implemented in pyAFQ, including checks for minimum streamline count, tract length, and anatomical plausibility. Reconstructions were visually inspected by a neurologist (D.Z.) to confirm anatomical plausibility and verify that tract trajectories aligned with expected neuroanatomical pathways.

### Analysis of direct tumor involvement

Left and right hemisphere homologues of each tract were averaged together for analysis, yielding 10 unique bilateral tract groups (e.g., left and right inferior longitudinal fasciculi analyzed as one tract group). A tract node was classified as tumor-involved if tumor components (enhancing tumor, necrotic core, or edema) occupied more than 5% of that node’s spatial extent. This threshold was chosen to provide objective criteria for distinguishing tumor-involved from unaffected tissue while maintaining sensitivity for subtle infiltrative changes, consistent with approaches in probabilistic tractography studies that demonstrate the importance of permissive thresholds in tumor environments [24, 25]. To assess robustness to this threshold choice, sensitivity analyses were performed using thresholds ranging from 1% to 15%, examining stability of key findings. Subject-level involvement was binarized as involved (≥1 node exceeding the 5% threshold) or not involved. Tract-level involvement was calculated as the proportion of nodes exceeding the 5% threshold within each tract.

### Analysis of tumor involvement beyond radiologic tumor margins

To compare white matter tissue properties in the hemisphere least affected by the tumor, we averaged the free water-corrected (FW-) FA, MD, and FWF across all nodes from the contralateral hemisphere for each patient. Then, to quantify the differences between the ipsilateral and contralateral white matter beyond the tumor margins, we computed a directional percent asymmetry at nodes with < 5% combined tumor involvement (enhancing tumor, necrotic core, and edema/infiltration), pairing nodes by index across hemispheres within the same tract:

$$\:Asymmetry\:\left(\%\right)\:=\:\frac{ipsilateral\:-\:contralateral}{(ipsilateral\:+\:contralateral)/2}\:\times\:\:100\%$$

Therefore, positive values indicated higher values in the ipsilateral white matter.

For whole-tract asymmetry comparisons, node-level asymmetries were averaged within the tract and then collapsed to a single subject-level mean per metric. Then, to test for peritumoral gradients, we computed the shortest along-tract node distance from each ipsilateral node to the nearest tumor edge, defined by any radiologic tumor component. Subject-level mean asymmetries were then compared within distance segments of five nodes (1–5, 6–10, 11–15, 16–20, and ≥ 21 nodes from the tumor edge), yielding one value per segment, metric, and subject.

### Statistical analysis

Analyses were conducted in Python (pandas, NumPy, SciPy, statsmodels, matplotlib). All statistical tests were two-sided, comparing imaging measures across pathology types (glioblastoma, astrocytoma, oligodendroglioma). Continuous variables were reported as median (IQR) or mean ± SD, and categorical variables as counts (percent).

Normality was assessed with Shapiro-Wilk tests. Three-group comparisons used: (1) Kruskal-Wallis for overall significance; (2) pairwise Mann-Whitney U tests; (3) linear or logistic regression with dummy coding (glioblastoma reference), adjusted for age and sex where appropriate. Multiple comparisons were controlled using Benjamini-Hochberg false discovery rate (FDR), stratified by test family. Statistical significance was denoted as *p* < 0.05 for individual tests, q < 0.05 for FDR-corrected comparisons within test families.

Tumor proportions were calculated as volume normalized to total brain volume, excluding ventricles. FWF-edema correlation used Spearman’s coefficient. Direct tract involvement used logistic regression for binary outcomes and ordinary least-squares (OLS) regression for the proportion of involved nodes. Contralateral hemisphere comparisons used linear regression adjusted for age and sex. Whole-tract and distance-based asymmetry analyses (1–5, 6–10, 11–15, 16–20, ≥ 21 nodes from margins) used Kruskal-Wallis and OLS regression without age/sex covariates since asymmetry is computed within subjects.

## Results

### Demographic and clinical features

The UCSF-PDGM dataset contained preoperative MRI scans from 495 unique patients, of whom 459 (93%) were included in the study. Of the excluded patients, 24 (5%) had tumors classified as Not Elsewhere Classified due to incomplete molecular testing, and 12 (2%) had bilateral white matter tract involvement, precluding hemispheric asymmetry analyses. Of the patients included, 361 (79%) were classified as glioblastoma, 87 (19%) as astrocytoma, and 11 (2%) as oligodendroglioma; mean age at time of MRI scan was 57 years (range 19–97), and 184 (40%) were female (Table 1). Patients with glioblastoma were significantly older than patients with astrocytoma or oligodendroglioma (both *p* < 0.001). No significant differences in sex distribution were observed among groups (*p* = 0.94). Glioblastoma had significantly larger enhancing tumor and necrotic core volumes compared to astrocytomas and oligodendrogliomas (both *p* < 0.001).

**Table 1:** Demographic and clinical comparisons

	Characteristic	Glioblastoma (*n* = 361)	Astrocytoma (*n* = 87)	Oligodendroglioma (*n* = 11)	*p*-value
Demographic features					
	Age, median (IQR)	62 (55–71)	36 (31-44.5)	45 (34.5–52)	< 0.001
	Female, n (%)	146 (40%)	34 (39%)	4 (36%)	0.94
Tumor grade					
	Low-Grade (2), n (%)	0	33 (38%)	9 (82%)	< 0.001
	High-Grade (3–4), n (%)	361 (100%)	54 (62%)	2 (18%)	< 0.001
Tumor proportion of total brain volume					
	Enhancing tumor, median (IQR)^a^	0.006 (0.002–0.016)	0 (0-0.0005)	0 (0–0)	< 0.001
	Necrotic core, median (IQR)^b^	0.016 (0.008–0.026)	0 (0-0.003)	0 (0–0)	< 0.001
	Edema/infiltration, median (IQR)	0.044 (0.022–0.071)	0.053 (0.030–0.100)	0.024 (0.015–0.036)	0.001
	Total tumor, median (IQR)	0.073 (0.040–0.110)	0.059 (0.030–0.112)	0.025 (0.015–0.036)	< 0.001

Abbreviations: IQR, interquartile range. ^a^ 34% of astrocytomas and 9% of oligodendrogliomas had detectable enhancement. ^b^ 47% of astrocytomas and 36% of oligodendrogliomas had detectable necrosis

### Tractography quality with free water elimination

**Fig. 2:**
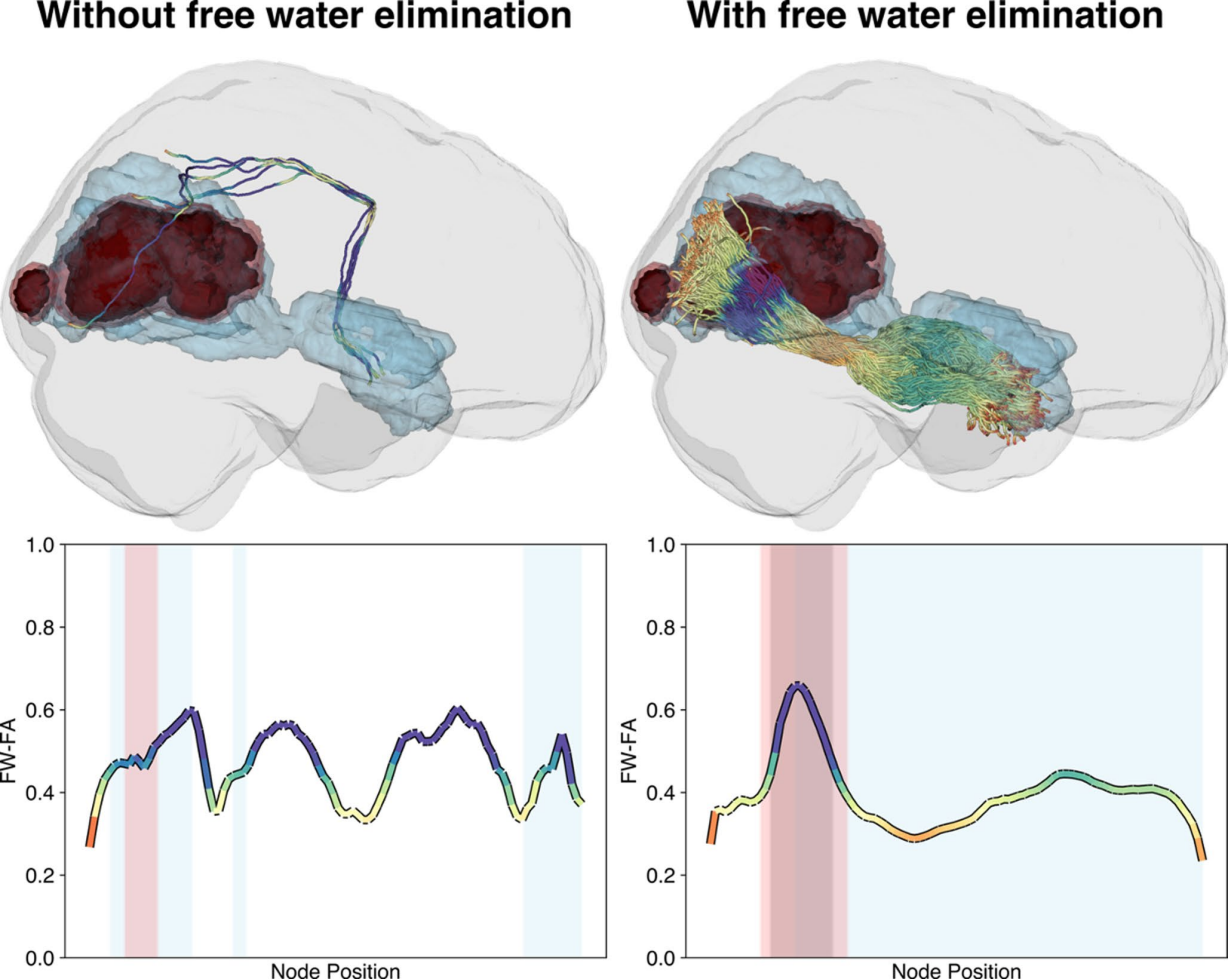
Representative example demonstrating improved tractography reconstruction with free water elimination. Comparison of the right inferior longitudinal fasciculus tractography results without (left) versus with (right) free water elimination for a representative glioblastoma patient (Subject #393, UCSF-PDGM dataset). The top panels show 3D reconstructions of white matter tracts overlaid on tumor segmentations (red: enhancing tumor, light blue: edema). Without free water elimination, only sparse tracts were successfully reconstructed in tumor-affected regions. With free water elimination, multiple major white matter bundles were successfully generated. Bottom panels show corresponding tract profiles of free water-corrected fractional anisotropy (FW-FA) along node position, with colored regions indicating tumor component involvement (red: enhancing tumor, blue: edema). Free water elimination enabled successful tract reconstruction through edematous regions where conventional tractography failed, demonstrating the methodological advantage for analyzing white matter in tumor environments

The white matter tracts were successfully reconstructed bilaterally and passed automated quality control procedures implemented in pyAFQ. Visual inspection of tractography with and without FWE revealed improved tract reconstruction in tumor-affected regions, with FWE enabling tract generation through edematous areas where conventional DTI showed sparse or failed reconstructions (representative example in Fig. 2). Free water fraction correlated strongly with edema involvement (ρ = 0.55, *p* < 0.001), suggesting that FWE successfully detected free water in edematous regions and enabling separation of tissue-specific diffusion from free water contamination (Supplementary Fig. S1E).

### Direct tumor involvement of white matter tracts

Quantitative analyses of white matter tract involvement revealed significant pathology-specific differences (Table 2). Across all white matter tracts, glioblastoma showed a higher mean proportion of tumor-involved nodes than astrocytoma and oligodendroglioma for enhancing tumor (5.4% vs. 1.3% vs. 0.0%, *q* < 0.001), necrotic core (1.7% vs. 0.7% vs. 0.0%, *q* = 0.01), and combined tumor components (17.3% vs. 15.3% vs. 5.8%, *p* = 0.001). Edema/infiltration involvement was substantial across all pathology types but remained significantly higher in glioblastoma and astrocytoma compared to oligodendroglioma (16.0% vs. 15.1% vs. 5.8%, *q* = 0.006). Sensitivity analyses testing tumor involvement thresholds from 1% to 15% demonstrated that these pathology-specific patterns remained stable across the threshold range (Supplementary Fig. S2).

**Table 2:** Proportion of Tumor-Involved nodes in white matter by pathology

Tumor component	Glioblastoma, mean ± SD	Astrocytoma, mean ± SD	Oligodendroglioma, mean ± SD	adj *p*-value	fdr *q*-value
Combined	0.173 ± 0.094	0.153 ± 0.097	0.058 ± 0.052	0.001	-
Enhancing tumor	0.054 ± 0.046	0.013 ± 0.030	0.000 ± 0.001	< 0.001	< 0.001
Necrotic core	0.017 ± 0.030	0.007 ± 0.020	0.000 ± 0.000	0.01	0.01
Edema/infiltration	0.160 ± 0.094	0.151 ± 0.095	0.058 ± 0.052	0.005	0.006

Abbreviations: FDR, false discovery rate. SD, standard deviation

Examination of direct tract involvement patterns at the subject level revealed pathology-dependent differences (Supplementary Fig. S3-S4). For enhancing tumor involvement, glioblastoma showed widespread tract involvement compared to astrocytoma, with the most pronounced differences in the inferior fronto-occipital fasciculus (56% vs. 20% of subjects, *q* < 0.001), arcuate fasciculus (45% vs. 9%, *q* < 0.001), and inferior longitudinal fasciculus (40% vs. 8%, *q* < 0.001). Necrotic core involvement followed similar patterns, with glioblastoma showing significantly higher rates in the arcuate fasciculus (15% vs. 3%, *q* = 0.01), inferior fronto-occipital fasciculus (21% vs. 8%, *q* = 0.009), and inferior longitudinal fasciculus (15% vs. 2%, *q* = 0.01) compared to astrocytoma.

### Remote white matter tissue properties

FW-corrected FA, MD, and FWF values were calculated for each tract node to assess white matter tissue properties in regions not directly inside the tumor. Distributional analysis revealed significant normality deviations for MD asymmetry (Shapiro-Wilk *p* < 0.001), justifying the application of non-parametric statistical approaches for unadjusted analyses. Analysis of the contralateral hemisphere to the tumor revealed no significant differences in any diffusion metric after adjusting for age and sex (Supplementary Table S1),.

Whole-tract asymmetry analysis of all nodes without tumor involvement revealed pathology-specific patterns (Supplementary Table S2). Oligodendroglioma demonstrated significantly higher FA asymmetry (+ 2.0 ± 2.2%) compared to glioblastoma (-3.0 ± 7.1%, *q* = 0.008) and astrocytoma (-2.2 ± 6.7%, *q* = 0.04). Both glioblastoma and astrocytoma showed negative FA asymmetry and positive MD asymmetry, indicating lower anisotropy and higher diffusivity in the ipsilateral hemisphere. In contrast, oligodendroglioma showed positive FA asymmetry and negative MD asymmetry, suggesting relatively preserved white matter microstructure in the tumor-bearing hemisphere. No significant differences were observed for FWF across any pathology comparisons.

**Fig. 3:**
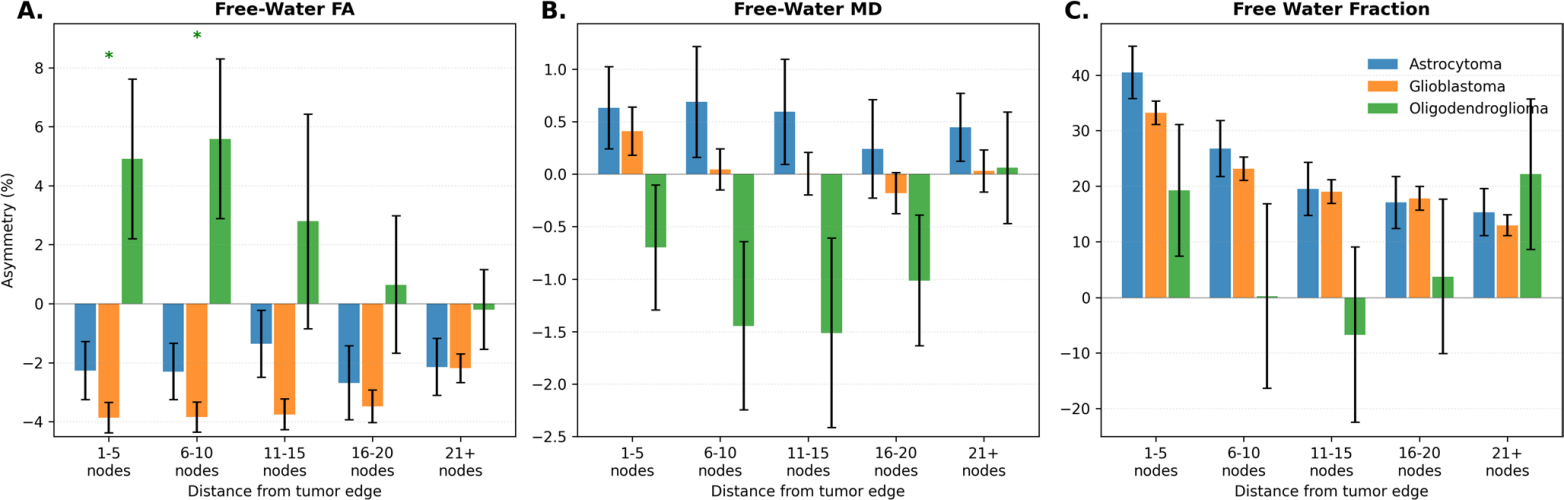
Distance-based white matter asymmetry index. For each tract, subject-level mean asymmetries of free-water corrected diffusion metrics, including fractional anisotropy (FW-FA), mean diffusivity (FW-MD), and free water fraction (FWF) were grouped by distance from the tumor margin (1–5, 6–10, 11–15, 16–20, and 21 + nodes). Unadjusted pairwise comparisons used Mann-Whitney U tests between pathology groups (Astrocytoma, Glioblastoma, Oligodendroglioma). Bars represent group means with error bars as the standard error of the mean. False discovery rate (FDR) correction was applied separately within each diffusion measure, creating 3 independent correction families for pairwise Mann-Whitney U tests. Green asterisks (*) denote distance bins with significant pairwise differences between glioblastoma and oligodendroglioma at q < 0.05 after FDR correction

Distance-based asymmetry analysis revealed pathology-dependent patterns at specific distances from radiologic tumor margins (Fig. 3). Oligodendroglioma demonstrated significantly higher FA asymmetry compared to glioblastoma at 1–5 nodes (+ 4.9 ± 9.0% vs. -3.9 ± 9.8%, *q* = 0.04) and 6–10 nodes (+ 5.6 ± 9.0% vs. -3.8 ± 9.7%, *q *= 0.03). The magnitude of this preservation effect diminished with increasing distance from tumor margins, with no significant differences observed beyond 10 nodes. No significant differences were observed between astrocytoma and glioblastoma at any distance, or for MD or FWF across any pathology comparisons.

## Discussion

This study demonstrates how applying open-source analytical methods to a large publicly available clinical imaging repository enables detection of white matter alteration patterns, allowing reproducible investigation of tumor biology. Glioblastoma showed the most extensive direct white matter involvement across contrast-enhancing and necrotic components, reflecting its aggressive properties and extensive invasion along white matter pathways. Astrocytoma showed intermediate involvement patterns, with lower enhancing tumor and necrotic core involvement than glioblastoma but more extensive involvement than oligodendroglioma. Beyond visible tumor margins, oligodendroglioma demonstrated positive FA asymmetry and negative MD asymmetry compared to both glioblastoma and astrocytoma, most pronounced within the proximal 10% of tract length from radiologic tumor margins, suggesting that oligodendroglioma’s slower growth and less infiltrative behavior result in less alteration of adjacent white matter. In contrast, both glioblastoma and astrocytoma showed negative FA asymmetry and positive MD asymmetry, consistent with microstructural alterations in the tumor-bearing hemisphere even beyond radiologically visible boundaries. These distinct patterns may inform presurgical risk assessment and radiation treatment planning, as peritumoral tract alterations could influence functional outcomes and guide therapeutic targeting beyond visible tumor margins.

While FWE isolated tissue-specific diffusion from free water contamination, the observed microstructural changes could reflect several processes: subclinical tumor infiltration beyond MRI detection limits, white matter deformation from mass effect, alterations in the tissue microenvironment from peritumoral processes, or secondary network effects from tract disruption. Although our cross-sectional imaging data did not distinguish among these possibilities, the distance-dependent gradient, where tract alteration was most pronounced proximally and attenuated with distance from tumor margins, raises the possibility that these signatures may capture spatial gradients of tumor infiltration. If these patterns reflect occult infiltrative extent, they could potentially inform estimates of subclinical disease burden relevant to surgical planning and recurrence risk. Validating this hypothesis would require prospective studies correlating preoperative tractometry findings with histopathological analysis of tissue sampled beyond radiographic margins and longitudinal mapping of recurrence patterns.

Our finding of no significant contralateral hemisphere differences contrasts with prior DTI studies reporting increased MD and reduced FA in the contralateral hemisphere [26, 27]. This discrepancy may reflect differences in patient populations, analysis methodologies, or our application of FWE techniques to better isolate tissue-specific diffusion properties. While the absence of contralateral effects could reflect limited statistical power or true sparing of the contralateral hemisphere, it suggests that the ipsilateral asymmetries are not fully attributable to global bilateral processes, supporting the spatial specificity of our distance-dependent findings.

FWE tractometry addressed key limitations of conventional DTI in tumor environments. This correction is particularly important in glioma imaging, where vasogenic edema and infiltration confound standard DTI metrics [14–16]. By isolating tissue microstructure from free water effects, FWE enabled more valid comparisons of white matter integrity across pathology types with different edema burdens. The strong correlation between FWF and edema validates the methodological approach and supports the biological interpretability of our tissue-specific measurements.

The study has several limitations that should be considered. The cross-sectional design precludes assessment of temporal changes in white matter properties or correlation with postoperative outcomes such as survival or functional status. The UCSF-PDGM dataset represents a single-institution cohort with specific imaging protocols, which may limit generalizability to other populations or scanners. However, the standardized protocols within this cohort enabled rigorous quantitative comparison without confounding scanner effects, a key advantage over heterogeneous multi-center datasets. Our exclusion of patients with bilateral or midline tumors, while necessary for proper asymmetry analysis, could introduce selection bias toward more focal lesions limiting generalizability to bilateral tumors. As the dataset was curated prior to the 2021 WHO molecular classification, we excluded cases with incomplete molecular testing to ensure diagnostic accuracy, reducing the sample sizes for some pathology comparisons.

Our use of single-shell data for FWE had inherent limitations compared to multi-shell acquisition. While FWE can be estimated from single-shell data with appropriate modeling constraints, multi-shell protocols provide improved precision in separating tissue and free water compartments, particularly in regions with complex partial volume effects [23]. The spatial regularization constraints required for single-shell FWE may reduce sensitivity to subtle tissue changes, potentially contributing to null findings for some metrics such as FWF [23, 28]. Nevertheless, all reconstructed tracts passed automated quality control in pyAFQ and visual inspection. These permissive thresholds may have retained some marginal reconstructions in large tumor cases, but avoided inappropriately excluding valid tracts. We also did not systematically assess whether tumor anatomical distribution differed across pathology subtypes in our cohort. Accurate lobar parcellation in the presence of mass effect and anatomical distortion presents significant methodological challenges and would require different imaging analysis approaches than those used in this diffusion-based tractometry study. A prior population-based study found no statistically significant differences in anatomical location between glioblastoma, astrocytoma, and oligodendroglioma, though we cannot exclude the possibility that distributional differences could contribute to the tract-specific involvement patterns observed in our cohort [29].

The oligodendroglioma cohort was substantially smaller than glioblastoma and astrocytoma groups, limiting statistical power for this subtype. Negative findings involving oligodendroglioma should be interpreted cautiously as they may reflect insufficient power rather than true absence of effect. Adequately powered studies would be needed to definitively characterize oligodendroglioma-specific patterns. Moreover, the 5% threshold for defining tumor involvement, while validated through sensitivity analyses across multiple thresholds, remains a methodological choice, and the optimal threshold for distinguishing tumor-involved from unaffected tissue in tractography studies has not been established in the literature. Finally, our imaging-based findings lacked clinical data such as functional outcomes, cognitive assessments, or histopathological validation of white matter involvement patterns, limiting the ability to establish clinical correlations of the observed diffusion changes.

This study demonstrates how a large-scale, publicly available clinical imaging dataset can reveal pathology-specific patterns of white matter alterations that could inform tumor biology and clinical decision-making. By applying FWE tractometry to the UCSF-PDGM cohort, we established a reproducible quantitative framework that addresses key limitations of conventional DTI in tumor environments and can be applied across institutions using standardized diffusion protocols. The pathology-specific patterns observed could provide imaging-based correlates of known biological differences with potential implications for surgical and radiation therapy planning. The open-source, reproducible methodology established here exemplifies how well-curated imaging repositories enable methodological innovation and biological discovery in neuro-oncology. Future integration of these tract-based imaging metrics with longitudinal clinical data from cancer registries and prospective surgical cohorts will validate their prognostic utility and advance their translation into clinical decision support tools. This work provides a foundation for multi-institutional efforts to standardize quantitative white matter assessment in glioma and demonstrates the continued value of investment in open neuroscience data infrastructure.

## Supplementary Information

Below is the link to the electronic supplementary material.

Supplementary Material 1

Supplementary Material 2

Supplementary Material 3

Supplementary Material 4

Supplementary Material 5

## Data Availability

The UCSF-PDGM dataset analyzed in this study is publicly available through The Cancer Imaging Archive (TCIA) at (10.7937/TCIA.2020.C1GQ4842) [10.7937/TCIA.2020.C1GQ4842] (10.7937/TCIA.2020.C1GQ4842). All tractography and tractometry analyses were performed using pyAFQ version 2.1, an open-source Python package available at (https://yeatmanlab.github.io/pyAFQ) [https://tractometry.org/pyAFQ/](https://tractometry.org/pyAFQ) and free-water elimination software available at [https://github.com/nrdg/fwe/] (https://github.com/nrdg/fwe). Results of tractometry analysis are available at: [https://figshare.com/articles/dataset/Free-water/_elimination/_tractometry/_from/_the/_The/_University/_of/_California/_San/_Francisco/_Preoperative/_Diffuse/_Glioma/_MRI/_dataset/30402736] (https://figshare.com/articles/dataset/Free-water_elimination_tractometry_from_the_The_University_of_California_San_Francisco_Preoperative_Diffuse_Glioma_MRI_dataset/30402736). Code to reproduce the statistical analysis and visualizations in the paper is available at [https://github.com/nrdg/fwe/_tractometry/_glioma] (https://github.com/nrdg/fwe_tractometry_glioma).
