# Supplementary figures and images for: Free water elimination tractometry reveals local and remote white matter alterations in diffuse gliomas
Source: J Neurooncol. 2025 Dec 10;176(1):115. doi: 10.1007/s11060-025-05370-w (PMC12696142; doi:10.1007/s11060-025-05370-w)

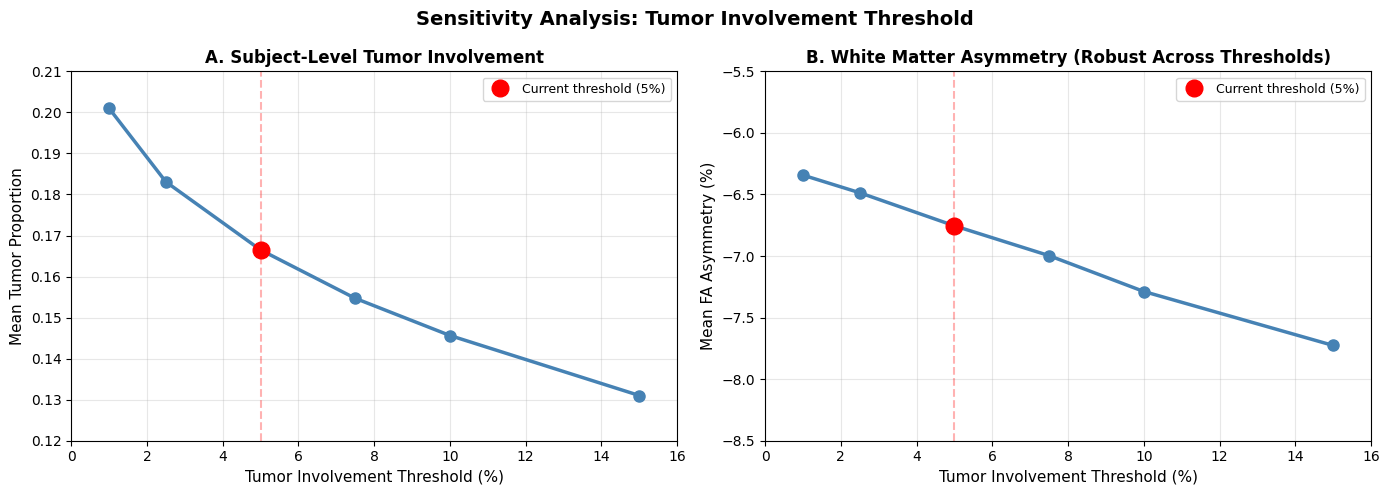

Supplement: Supplementary file 1 — Supplementary Material 1 [file 11060_2025_5370_MOESM1_ESM.png]

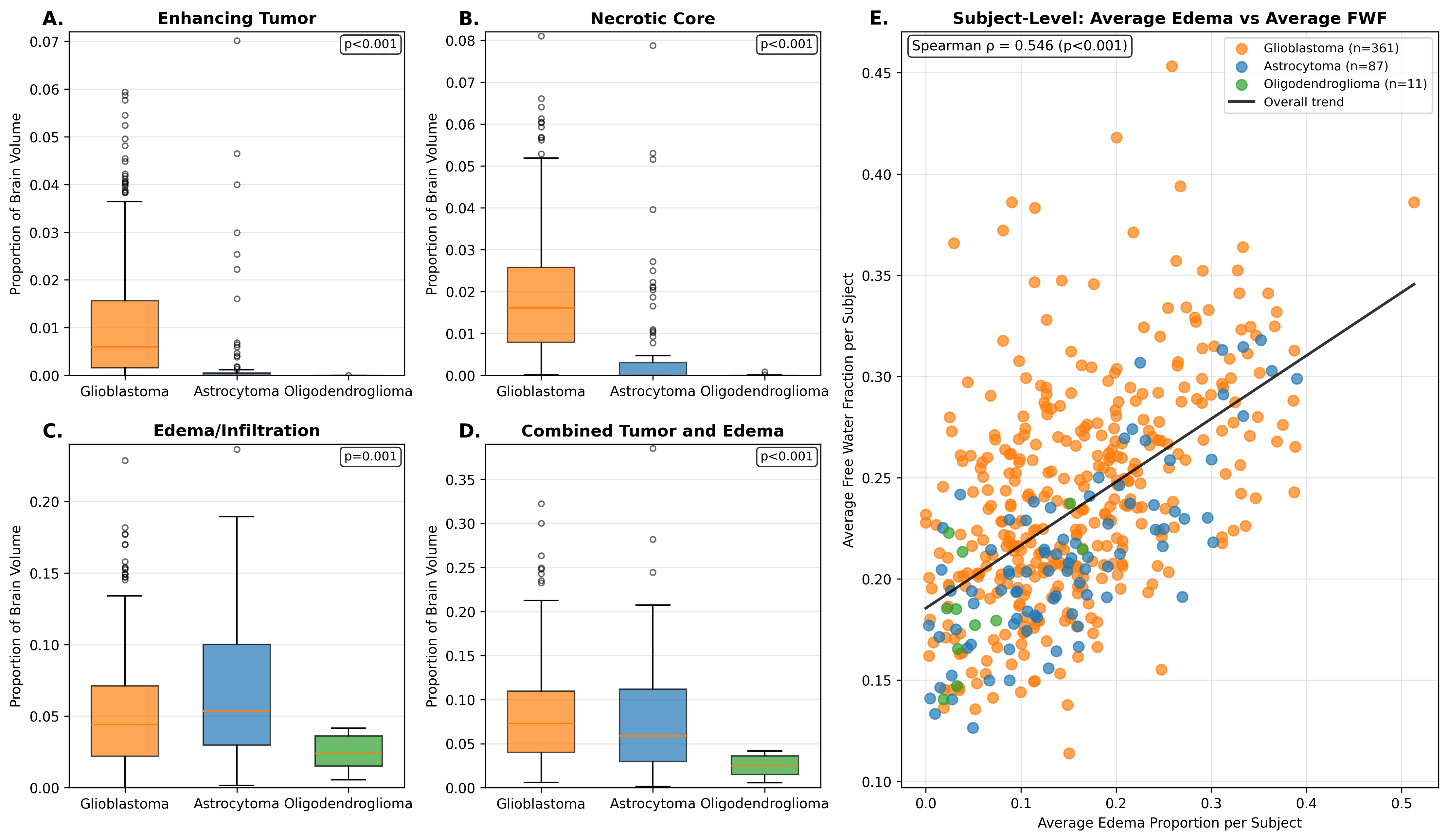

Supplement: Supplementary file 2 — Supplementary Material 2 [file 11060_2025_5370_MOESM2_ESM.png]

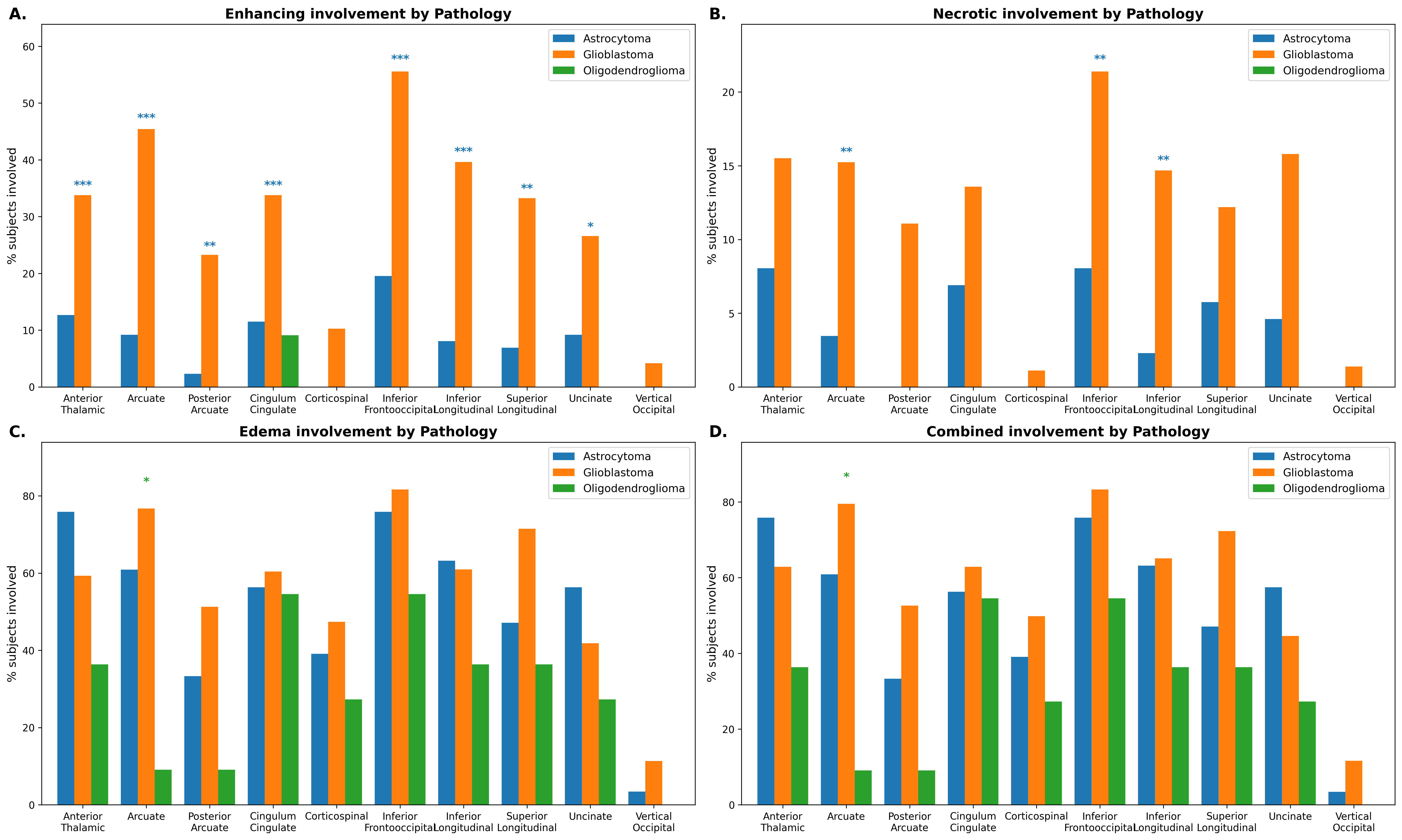

Supplement: Supplementary file 3 — Supplementary Material 3 [file 11060_2025_5370_MOESM3_ESM.png]

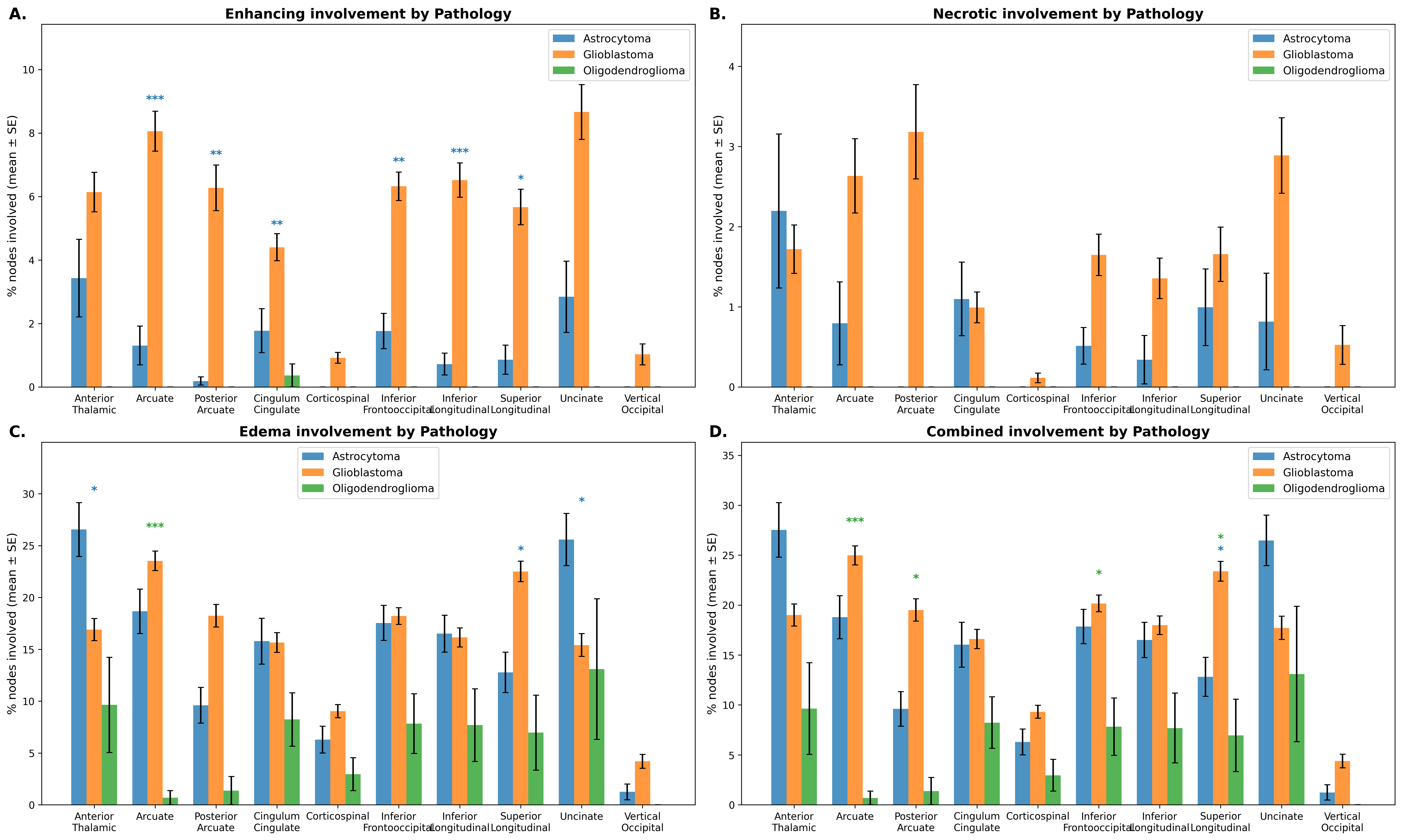

Supplement: Supplementary file 4 — Supplementary Material 4 [file 11060_2025_5370_MOESM4_ESM.png]
