# Supplementary material for: Free water elimination tractometry reveals local and remote white matter alterations in diffuse gliomas
Source: J Neurooncol. 2025 Dec 10;176(1):115. doi: 10.1007/s11060-025-05370-w (PMC12696142; doi:10.1007/s11060-025-05370-w)
Supplement: Supplementary file 5 — Supplementary Material 5 [file 11060_2025_5370_MOESM5_ESM.docx]

**Supplementary Tables and Figures**

**Table S1. Comparison of White Matter Diffusion Metrics in the Contralateral Hemisphere**

| **Tract measure** | **Glioblastoma, mean ± SD** | **Astrocytoma, mean ± SD** | **Oligodendroglioma, mean ± SD** | **p-value** | **q-value** |
| --- | --- | --- | --- | --- | --- |
| Beltrami-FA | 0.42 ± 0.02 | 0.43 ± 0.02 | 0.42 ± 0.02 | 0.07 | 0.21 |
| Beltrami-MD | 0.00060 ± 0.00001 | 0.00059 ± 0.00001 | 0.00060 ± 0.00001 | 0.44 | 0.49 |
| Beltrami-FWF | 0.21 ± 0.06 | 0.18 ± 0.04 | 0.18 ± 0.02 | 0.49 | 0.49 |

**Abbreviations:** FA, fractional anisotropy. FW, free water. FWF, free water fraction. MD, mean diffusivity. SD, standard deviation.

**Table S2. Comparison of Whole-Tract Hemispheric Asymmetry of White Matter Diffusion Metrics**

| **Tract measure** | **Glioblastoma, mean ± SD** | **Astrocytoma, mean ± SD** | **Oligodendroglioma, mean ± SD** | **Adj p-value** | **FDR q-value** |
| --- | --- | --- | --- | --- | --- |
| Beltrami-FA | -3.0 ± 7.1 | -2.1 ± 6.7 | 2.0 ± 2.2 | 0.01 | 0.02 |
| Beltrami-MD | 0.1 ± 2.8 | 0.4 ± 2.6 | -0.5 ± 1.5 | 0.74 | 0.74 |
| Beltrami-FWF | 18.7 ± 29.9 | 20.0 ± 31.2 | 14.9 ± 37.9 | 0.23 | 0.35 |

**Abbreviations:** FA, fractional anisotropy. FW, free water. FWF, free water fraction. MD, mean diffusivity. SD, standard deviation. Positive values represent a higher ipsilateral hemisphere value compared to the contralateral hemisphere value. The p-value was adjusted based on sex and age.


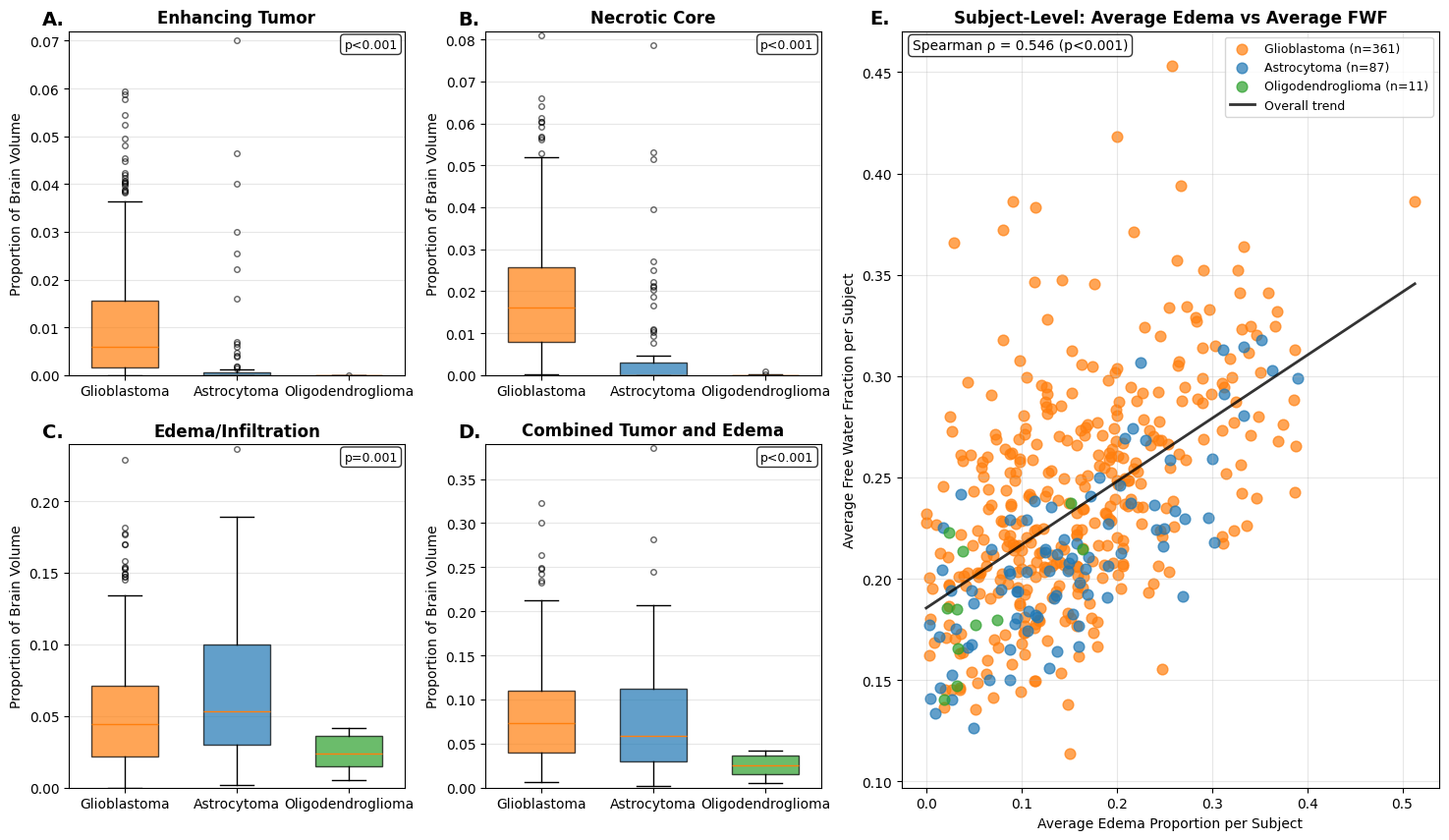


**Fig. S1** (A-D) Tumor component volumes by pathology. Box plots showing the proportion of brain volume occupied by different tumor components across three pathology types: Glioblastoma (orange), Astrocytoma (blue), and Oligodendroglioma (green). (A) Enhancing tumor volume. (B) Necrotic core volume. (C) Edema/infiltration volume. (D) Combined tumor and edema volume. Statistical comparisons used Kruskal-Wallis test for three-group comparisons, with p-value shown. (E) Subject-level edema proportion versus free water fraction relationship. Scatter plot showing the correlation between average edema proportion and average free water fraction per subject, colored by tumor pathology. Each point represents one subject's average across all white matter tracts. The black line shows the overall linear trend across all subjects. Spearman correlation coefficient (ρ) and significance level are displayed


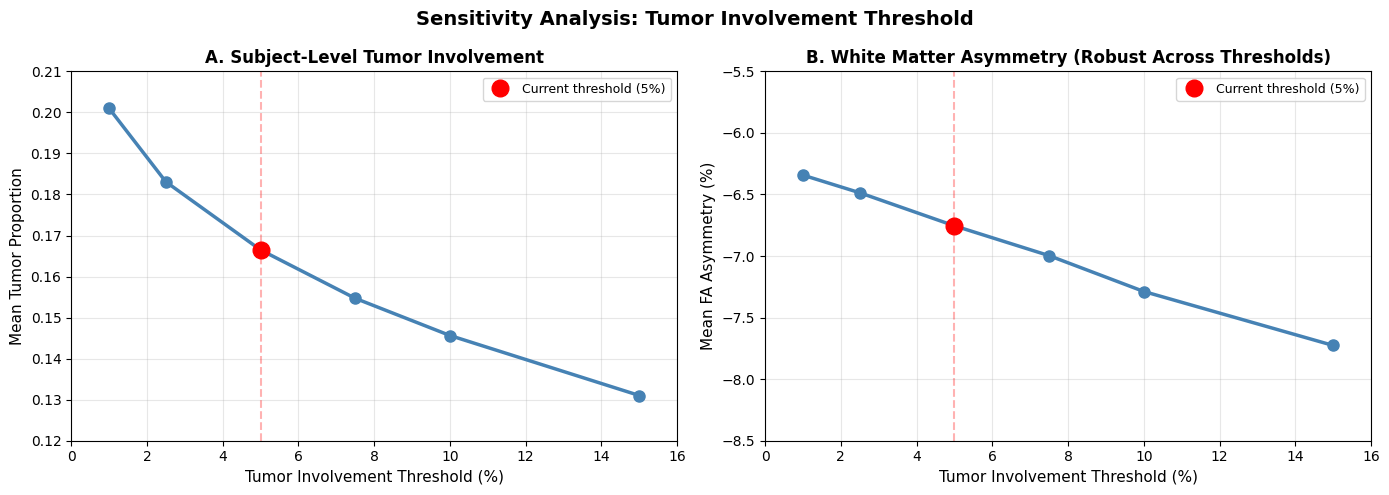


**Fig. S2.** The 5% tumor involvement threshold used to classify tract nodes was tested across a range of alternative thresholds (1%, 2.5%, 5%, 7.5%, 10%, and 15%). (A) Subject-level tumor involvement and (B) mean FA asymmetry across different tumor classification thresholds (1%-15%). The 5% threshold (red) balances classification specificity while maintaining stable asymmetry measurements, confirming robustness of findings to threshold selection


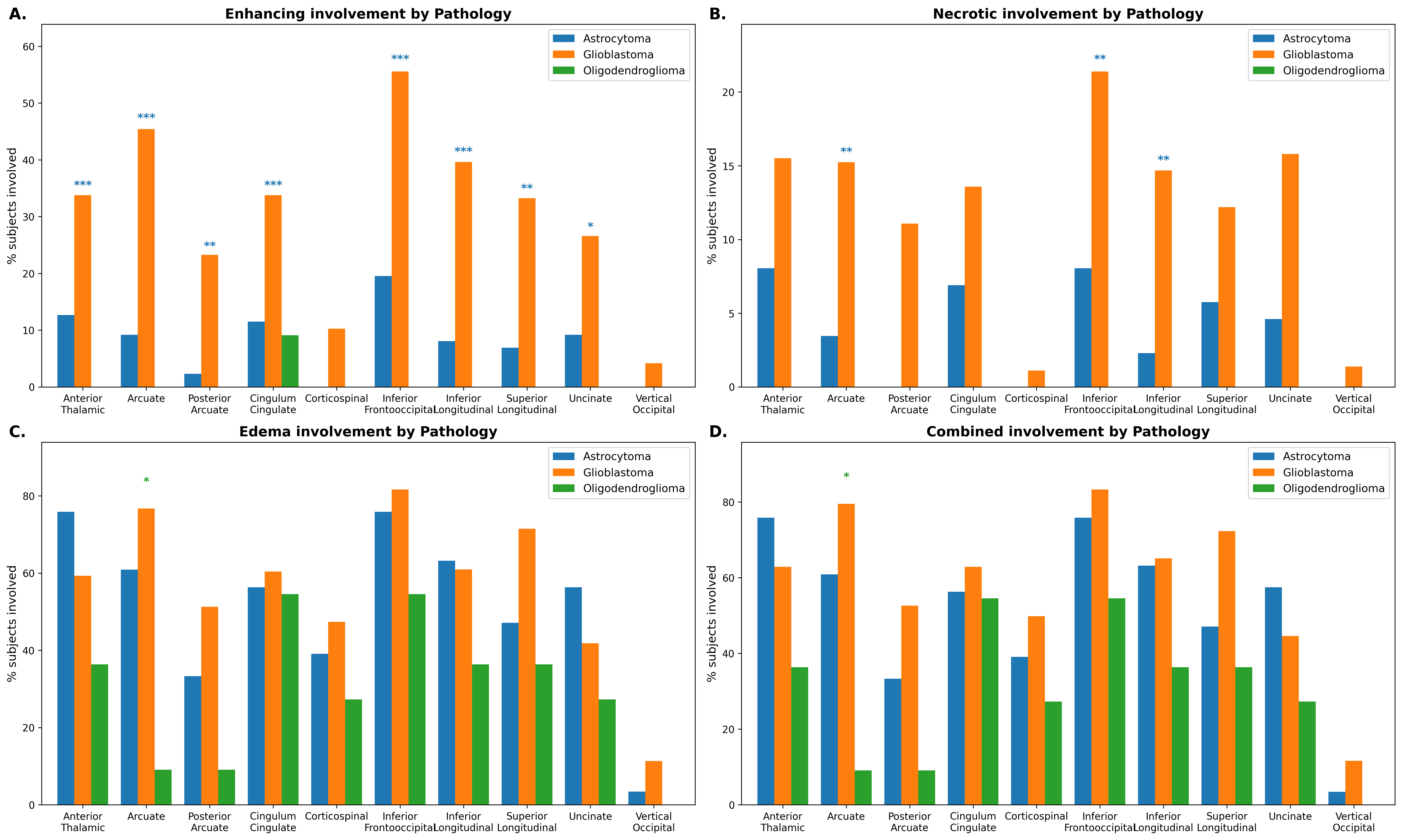


**Fig. S3** Percent of subjects with tract involvement (≥1 node with >5% tumor probability) by pathology type. (A) Enhancing tumor, (B) Necrotic core, (C) Edema, (D) Combined involvement across all tumor components. Pairwise comparisons between glioblastoma (reference) and astrocytoma, and between glioblastoma and oligodendroglioma were performed with logistic regression, adjusted for age and sex. *FDR-corrected p<0.05, **p<0.01, ***p<0.001. Blue and green asterisks (*) represent comparisons between glioblastoma and astrocytoma, and between glioblastoma and oligodendroglioma, respectively


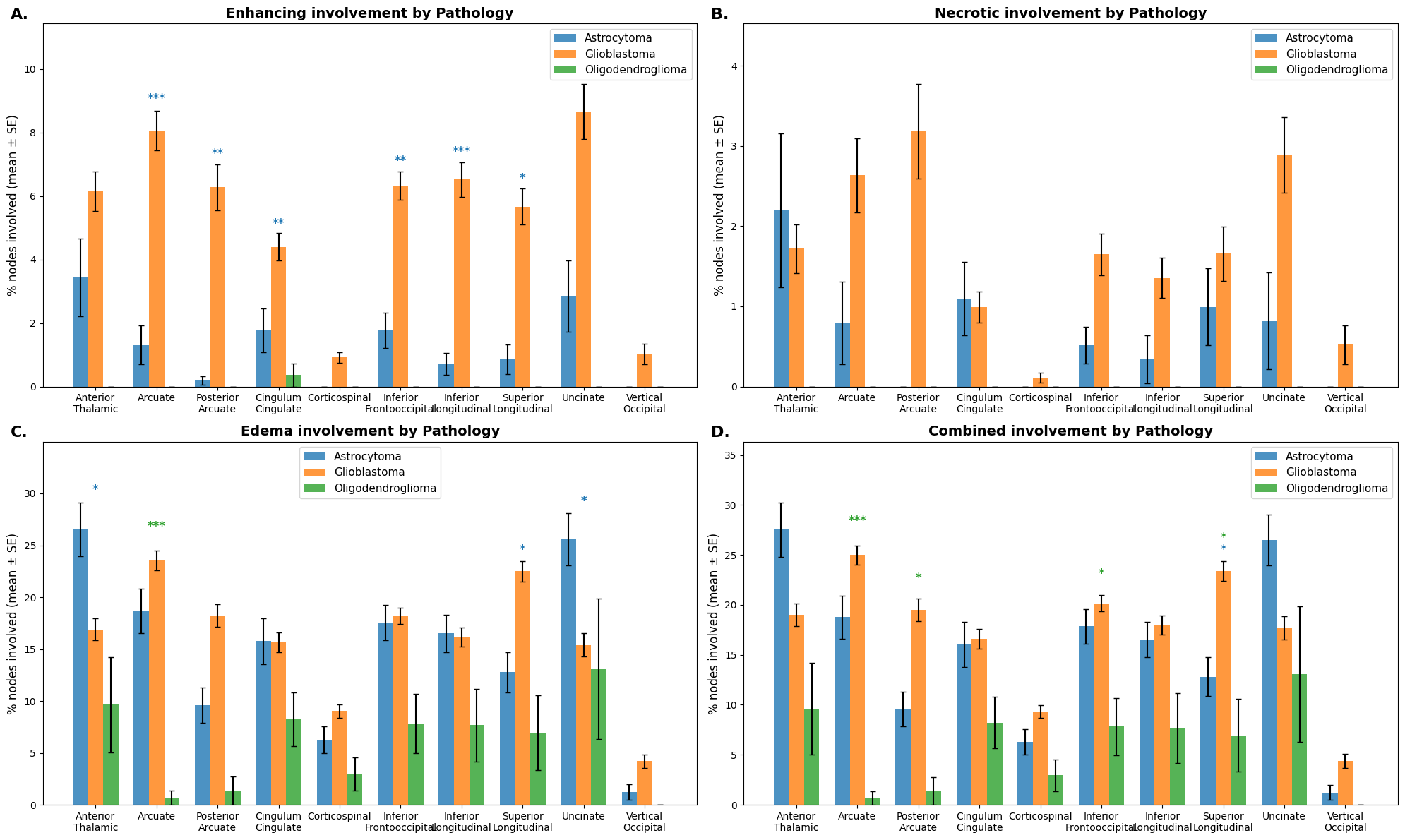


**Fig. S4.** Mean tract-level involvement (proportion of nodes >5%). (A-D) Mean proportion of nodes involved across 10 major white matter tracts for each tumor component (A) enhancing tumor, (B) necrotic core, (C) edema or infiltration, and (D) combined, stratified by pathology type. Error bars represent standard error of the mean. Statistical analysis used linear regression adjusted for age and sex, with separate FDR correction within each pairwise comparison and component family. Left and right tract segments were averaged to create bilateral tract measures. Colored asterisks indicate significant pairwise differences compared to Glioblastoma (reference group) after FDR correction: blue asterisks for Astrocytoma vs Glioblastoma, green asterisks for Oligodendroglioma vs Glioblastoma (*q < 0.05, **q < 0.01, ***q < 0.001)
